# Delayed recognition of autism spectrum disorder and attention-deficit/hyperactivity disorder in a girl with ornithine transcarbamylase deficiency: A case report

**DOI:** 10.1097/MD.0000000000033055

**Published:** 2023-02-22

**Authors:** Shin Kadono, Dai Miyawaki, Ayako Goto, Kaoru Hirai, Shoko Sakamoto, Hiroki Hama, Sayaka Nishiura, Takashi Hamazaki, Koki Inoue

**Affiliations:** a Department of Neuropsychiatry, Osaka Metropolitan University (Osaka City University) Graduate School of Medicine, Abeno-ku, Osaka, Japan; b Department of Pediatrics, Osaka Metropolitan University (Osaka City University) Graduate School of Medicine, Abeno-ku, Osaka, Japan.

**Keywords:** attention-deficit/hyperactivity disorder, autism spectrum disorder, hyperammonemia, ornithine transcarbamylase deficiency, urea cycle disorder

## Abstract

**Rationale:**

Ornithine transcarbamylase (OTC) deficiency, a urea cycle disorder, is a rare congenital metabolic error that leads to hyperammonemia. Psychiatric symptoms of hyperammonemia are nonspecific and can cause autism spectrum disorder (ASD)-like symptoms and attention-deficit/hyperactivity disorder (ADHD)-like symptoms. Some studies report that OTC deficiency is often initially diagnosed as ASD or ADHD. However, there are no reports of OTC deficiency comorbid with ASD and ADHD.

**Patient concerns:**

The patient is 17-year-old girl diagnosed with OTC deficiency at 3 years of age. She had behavioral problems since childhood, including depressed mood, irritability, and impulsive behavior; however, they were considered OTC-mediated nonspecific psychiatric symptoms. Therefore, the patient had not been appropriately assessed for ASD and ADHD. She presented with depressed mood and self-harm at 17 years of age.

**Diagnoses:**

We diagnosed her with ASD and ADHD based on her medical history and semistructured interviews.

**Interventions:**

We focused her ASD and ADHD traits and discussed strategies with her for better adaptive living.

**Outcomes:**

Our interventions resulted in her better social adjustment.

**Lessons:**

Physicians should consider the possibility of comorbid ASD and ADHD in individuals with OTC, facilitating appropriate and intervention for better outcomes.

## 1. Introduction

Ornithine transcarbamylase (OTC) deficiency, an X-linked disorder caused by mutations in the OTC gene, results in elevated blood ammonia levels. Autism spectrum disorder (ASD) is a neurodevelopmental disorder characterized by impaired social communication and repetitive patterns of behaviors, frequently comorbid with attention-deficit/hyperactivity disorder (ADHD). Moreover, it is characterized by hyperactivity, impulsivity, and difficulty concentrating. Psychiatric symptoms of hyperammonemia are nonspecific, and several studies report case of patients with OTC deficiency, initially diagnosed with ASD or ADHD.^[1–4]^

We describe the case of a 17-year-old girl with ASD and ADHD combined with OTC deficiency. She was diagnosed and treated for OTC deficiency early in her development, but her psychiatric symptoms were considered OTC-mediated nonspecific psychiatric symptoms. Although OTC deficiency and ASD and ADHD can be comorbid, there are no reports of OTC deficiency comorbid with ASD, in addition to ADHD.

## 2. Case report

Here, we describe the case of a 17-year-old Japanese girl with OTC deficiency. She did not undergo screening tests for OTC deficiency, because it is not included in newborn screening tests in Japan. She was examined by a pediatrician at the age of 3 years because of gait disturbance and somnolence. Previously, she had been displaying tantrums, hitting and injuring other children, and breaking things. She had liver dysfunction and hyperammonemia. Following a detailed examination that detected elevated blood glutamine levels and an OTC gene mutation (IVS4-1 G > C heterozygous), she was diagnosed with OTC deficiency. She had no relevant family history of OTC deficiency. She was prescribed a low-protein diet, levocarnitine, l-arginine, and benzoic acid, resulting in her blood ammonia levels being normalized. Moreover, her gait disturbance and somnolence disappeared. She was examined using the Wechsler Intelligence Scale for Children-Third Edition at 7 years of age. The results were full-scale intelligent quotient, 88, verbal comprehension index, 95, and perceptual reasoning index, 88.^[5]^

Despite an early and appropriate diagnosis of OTC deficiency and stabilized ammonia levels for a prolonged period, she was often reprimanded by her teachers after entering elementary school for not following their instructions in class or for leaving a room without putting the toys away after playing. Moreover, she did not develop good friendships. She often refused to attend school after the age of 10. At 11 years of age, her parents divorced, with her mother obtaining custody. Subsequently, her access to medications became irregular and she could no longer adequately adhere to the dietary protein restrictions. Her blood ammonia levels increased to 80 to 90 μg/dL, which is above the normal range, and she was repeatedly admitted to the pediatric hospital for hyperammonemia. After entering junior high school, she gradually became depressed and was unable to attend school. The situation persisted after entering high school, and she began to engage in self-harm including arm cutting and self-choking. At 17 years of age, she was referred to our hospital by a pediatrician, who led her first visit to a psychiatrist.

We conducted a detailed interview of her life history and confirmed consistently impaired social communication, repetitive behavior patterns, hyperactivity, impulsivity, and difficulty concentrating, which persisted since her early childhood. We assessed her for comorbid ASD and ADHD. She displayed poor nonverbal communication skills. Moreover, she faced difficulties in developing and maintaining relationships with other children. For example, she occasionally responded to angry people by laughing, which made them angrier. She has had restricted and fixed interests, such as immersing herself only in a specific character in animation. Moreover, she displayed poor impulse control. These symptoms were consistent from childhood and persisted regardless of metabolic control. She had impaired social communication skills, restricted and repetitive behavioral patterns, and difficulty focusing. We assessed her symptoms using the Pervasive Developmental Disorders Autism Society Japan Rating Scale and Kiddie Schedule for Affective Disorders and Schizophrenia.^[6,7]^ Based on her medical history and information, she was diagnosed with ASD and ADHD.

We hypothesized that her depressed mood and self-harm were caused by the difficulty in social adjustment owing to her inability to interact with others, which was in turn caused by ASD and ADHD. During her visit to our hospital, her social and occupational functioning assessment scale (SOFAS) score was 35, indicating major impairment in school and friendships.^[8]^ We decided to educate her about ASD and ADHD and to follow-up on her neuropsychiatric symptoms. Her blood ammonia level ranged from 80 to 90 μg/dL at baseline. Occasionally, she responded to psychosocial events and became more depressed, leading to more frequent self-harm. Concurrently, she could not adequately adhere to the low-protein diet or medications, and her ammonia levels were further elevated. The pediatrician continued the treatment for hyperammonemia, and we monitored her neuropsychiatric symptoms. She was occasionally admitted to a psychiatric ward upon experiencing a particularly strong depressive episode. We discussed strategies with her for better adaptive living, considering her ASD and ADHD traits. We observed her failure to sufficiently understand the intended meaning communicated and discussed this observation with her. We aimed to help her develop more adaptive relationships. Consequently, we drafted subgoals for her daily life and structured the environment such that she could achieve them. For example, we visualized her daily schedule on a piece of paper and used the alarm function on her watch to remind her of her appointments.

After graduating from high school, she continued to receive outpatient psychiatric treatment and was able to secure a part-time job with employment support that considered her ASD and ADHD symptoms. We continued to provide her with supportive outpatient treatment, not medication, and her SOFAS score improved to 70, indicating slight impairment in social and occupational functioning.^[8]^ Written consent was obtained from the patient and her parents to present this case.

## 3. Discussion

Here, we reported a case of ASD and ADHD, comorbid with OTC deficiency. We deciphered 2 findings in this case. First, OTC deficiency can be comorbid with ASD and ADHD. Second, appropriate and timely interventions for ASD and ADHD traits in patients with comorbid OTC deficiency may improve their psychiatric symptoms, resulting in better long term prognosis.

Patients with urea cycle disorders present with psychiatric symptoms of varying severity, including neurocognitive, behavioral, attentional, and executive functioning deficits.^[9]^ In some cases, patients are initially diagnosed with ASD or ADHD and only later diagnosed with an inborn error of metabolism.^[1–4]^ Kim et al^[1]^ reported a case of the patient, initially diagnosed and treated for ADHD, subsequently diagnosed with OTC deficiency owing to hemiplegia. Moreover, her neuropsychiatric symptoms improved with the normalization of ammonia levels. Serrano et al^[2]^ reported the case of a boy with carbamoyl phosphate synthesis disorder, initially diagnosed with ASD, whose delayed language development and social skills improved with dietary therapy. Children with urea cycle disorders demonstrate cognitive deficits, despite mild symptoms; therefore, it is important to maintain meticulous metabolic control.^[10]^ In the case discussed by Niwinski et al^[3]^, the ASD traits in a patient improved after treatment for OTC deficiency, but her blood ammonia level remained around 200 μg/dL. In our case, the patient’s blood ammonia level was around 80 μg/dL, but her ASD and ADHD traits were consistently present. This suggests that in this case, hyperammonemia and neurodevelopmental disorders exist independently. However, there is limited evidence for the comorbidity of inborn metabolic errors and ASD or ADHD. In this case, we used a detailed history from childhood and semi structured interview responses to diagnose the comorbidity of ASD and ADHD with OTC deficiency. This novel report demonstrated OTC deficiency comorbidity with ASD and ADHD.

ASD can be complicated by various comorbidities, such as intellectual development, ADHD, and depression.^[11]^ Particularly, depression and anxiety are common comorbidities.^[12]^ Daviss et al^[13]^ reported that the rate of depression in youths with ADHD was higher than in youths without ADHD. Moreover, ADHD and ASD coexist at high rates.^[14]^ Patients with ASD complicated by ADHD or ADHD complicated by ASD exhibit more severe anxiety and depressive symptoms.^[15,16]^ This necessitates assessment of comorbid depression in ASD and ADHD. Furthermore, neurodevelopmental disorder comorbidities affect the treatment interventions for depression.^[11,13]^ In this case, depressive symptoms and self-harm were supposedly caused by difficulties in social adjustment. The patient, caregivers, and medical staff recognized the presence of ASD and ADHD traits as the trigger of psychiatric symptoms, leading to improvement in psychiatric symptoms and social prognosis. Moreover, the individualized treatment of ASD is likely to lead to employment.^[17]^ Routine, structured, and continuous support from trusted people is essential for maintaining occupational activities in patients with ADHD.^[18]^ Thus, considering neurodevelopmental disorders to improve psychiatric symptoms and social prognosis, is necessary. However, its role in comorbid metabolic diseases in patients with ASD and ADHD is unclear. In this case, we supported her employment activities following a better understanding of the traits of ASD and ADHD through disease education, leading to employment and improvement in SOFAS. These efforts were made in the presence of mild hyperammonemia as the baseline. The interventions for ASD and ADHD may be effective even during comorbid OTC deficiency.

## 4. Conclusions

Clinicians need to pay more attention to the comorbidity of ASD and ADHD in patients with OTC deficiency. Neurodevelopmental disorders-like symptoms in patients with OTC deficiency have been reported to be treatable by controlling hyperammonemia, leading to the possibility of underestimating the comorbid ASD and ADHD. The diagnosis of ASD and ADHD may improve the long term prognosis by allowing patients to take individualized measures based on their specific traits. Therefore, clinicians should consider the possibility of comorbid neurodevelopmental disorders when psychiatric symptoms and social adjustment do not improve with the appropriate management of urea cycle abnormalities. However, there is limited evidence for the effectiveness of therapeutic interventions for patients with ASD and ADHD with hyperammonemia-associated metabolic diseases, including OTC deficiency; this necessitates further research in this field.

## Acknowledgments

The authors thank the patient and her mother for the agreement to publish this study.

## Author contributions

**Writing – original draft:** Shin Kadono, Dai Miyawaki.

**Writing – review & editing:** Ayako Goto, Kaoru Hirai, Shoko Sakamoto, Hiroki Hama, Sayaka Nishiura, Takashi Hamazaki, Koki Inoue.
